# A Rare Case of Tibial Intraneural Ganglion Cyst Arising From the Tibiofibular Joint

**DOI:** 10.7759/cureus.13570

**Published:** 2021-02-26

**Authors:** Savannah L Mayer, Jagmeet S Grewal, Tyler Gloe, Catherine A Khasho, Steven Harder

**Affiliations:** 1 Family Medicine, Des Moines University, Des Moines, USA

**Keywords:** knee ganglion cyst, large multiloculated ganglion cyst, intraneuronal, tibial nerve, tibiofibular joint

## Abstract

Intraneural ganglion cysts are a rare occurrence. They are most commonly found originating from the common peroneal nerve but are also frequently reported on the radial, ulnar, median, sciatic, tibial, and posterior interosseous nerves. A typical clinical presentation is posterior knee and calf pain resulting from tibial neuropathy with preferential degeneration of the popliteus muscle. Symptoms include pain, paresthesias, and decreased strength that originates in the knee and commonly extends to the plantar surface of the foot. These findings can be mistaken for lumbar neuropathies and compression of the sacral nerve roots. Differential diagnosis includes peripheral nerve sheath tumors, Baker’s cysts, extraneural ganglion cysts, and atypical vascular or lymphatic malformations. In this case report, the patient was a 61-year-old male, previously in good health, who presented with progressive pain in his medial left hamstring as well as weakness in left foot plantar flexion and paresthesias in the plantar aspect of his left foot. He first noticed impairments with his ability to push off with his left foot when running. His electromyogram (EMG) was abnormal and subsequent MRI of the left leg showed a complex intraneural ganglion cyst arising from the tibiofibular joint and ascending into the tibial nerve. He underwent indirect decompression through joint resection. Unfortunately, he did not have clinical improvement on one-year follow-up. Overall, symptomatic treatment of intraneural ganglion cyst includes decompression, surgical excision, or minimally invasive decompression by percutaneous aspiration of the ganglion under ultrasound guidance.

## Introduction

Ganglion cysts are non-neoplastic synovial or gelatinous cysts primarily consisting of hyaluronic acid, which originate within the epineurium of peripheral nerves. Although most ganglion cysts are asymptomatic, cysts arising in the peripheral nerves can lead to pain, paresthesias, pressure, and decreased mobility [1-2]. Cysts originating from the tibial nerve are rare and account for less than 15 cases in recent literature. Clinical manifestations can be sudden or gradual, most often precipitated by trauma to the region. Advancements in ultrasonography have made the diagnosis and treatment of non-complex ganglion cysts relatively simple, but more complex ganglion cysts with articular branches require surgery [2-3]. In this article, we will discuss a case of tibial intraneural ganglion cyst arising from the tibiofibular joint.

## Case presentation

This study was conducted in accordance with the ethical standards of the Institutional Review Board of our institution, Des Moines University College of Osteopathic Medicine.

A 61-year-old male patient presented with progressive pain in his medial left hamstring as well as weakness with left-sided foot drop and paresthesias in the plantar aspect of his foot. Symptoms first presented as impairments in his ability to push off with his left foot during moderate exercise. He did not present with radicular symptoms but did have a previous X-ray of the lumbar spine, which showed no signs of stenosis or compression.

Upon initial physical examination, the patient was found to have no tenderness to palpation of his lumbar spine. Tenderness was appreciated in the medial left glute and left posterior medial thigh down to the knee. No reproducible pain was noted below the knee, and no obvious foot deformity, erythema, or edema were present. He did endorse decreased sensation in the distal third of the plantar foot including the first metatarsal. The patient was unable to flex or extend his left first metatarsal. Left Achilles tendon reflex was 0/5, with a contralateral reflex of 5/5.

The patient was subsequently evaluated by a physical medicine and rehabilitation specialist, who noted diminished left S1 reflex. He was able to stand in plantarflexion on the right, but was unable to flex to full height on the left. Also, weakness of the left flexor hallucis longus and digitorum longus with absent sensation on the plantar surface of the first metatarsal to pinprick and light touch was noted. He was then referred for osteopathic manipulative therapy (OMT). Initial treatments with OMT did offer some relief, but his symptoms persisted. An electromyogram (EMG) was performed, which showed evidence of peroneal nerve injury in the absence of a superficial peroneal sensory response, temporal dispersion when stimulating across the knee, and tibial nerve injury.

**Figure 1:**
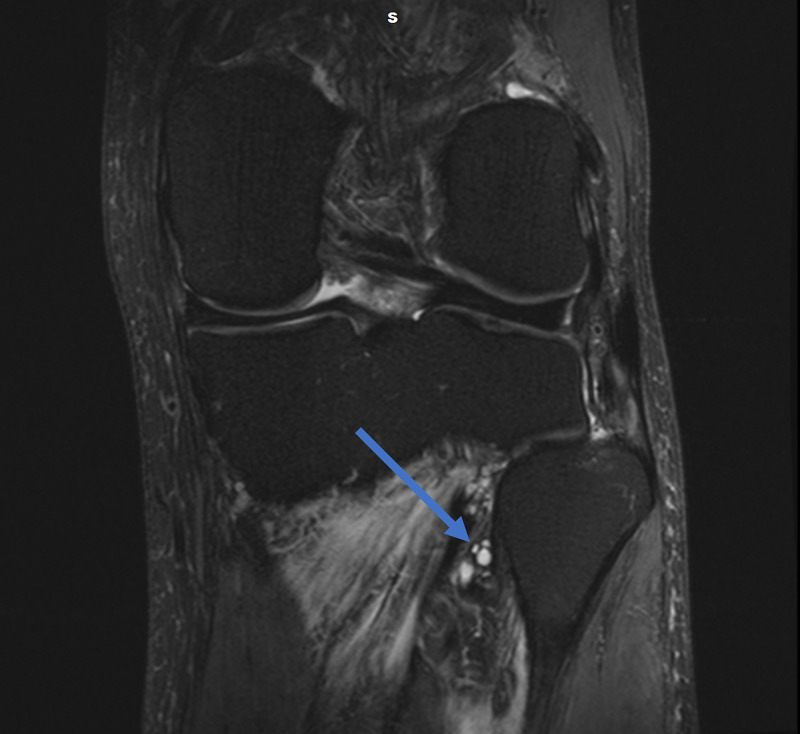
Coronal T2-weighted TSE fat-saturated image demonstrating cysts connecting to the posteromedial superior tibiofibular joint TSE, turbo spin echo

**Figure 2:**
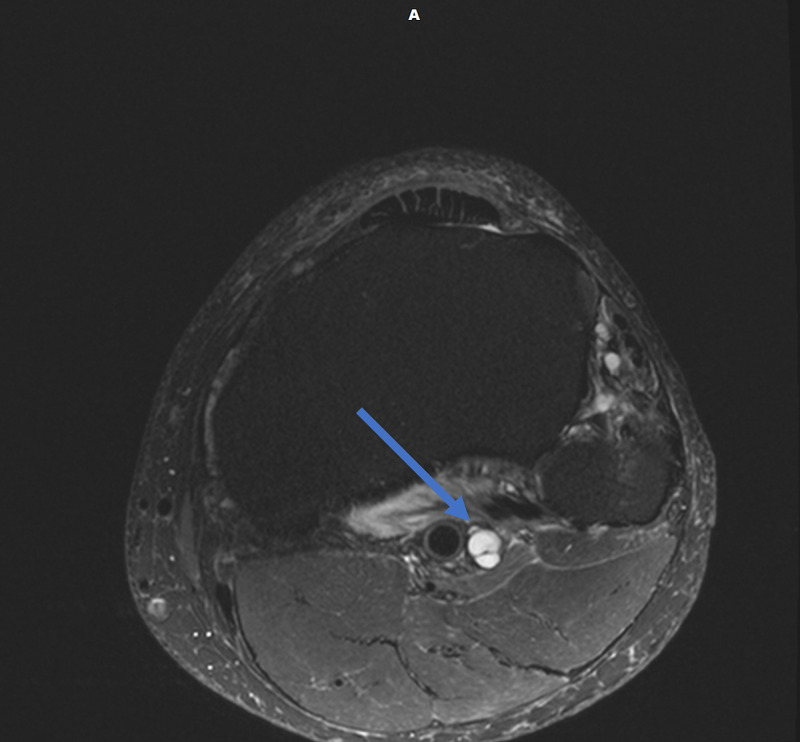
Axial T2-weighted TSE fat-saturated image demonstrating cysts connecting to the posteromedial superior tibiofibular joint TSE, turbo spin echo

**Figure 3:**
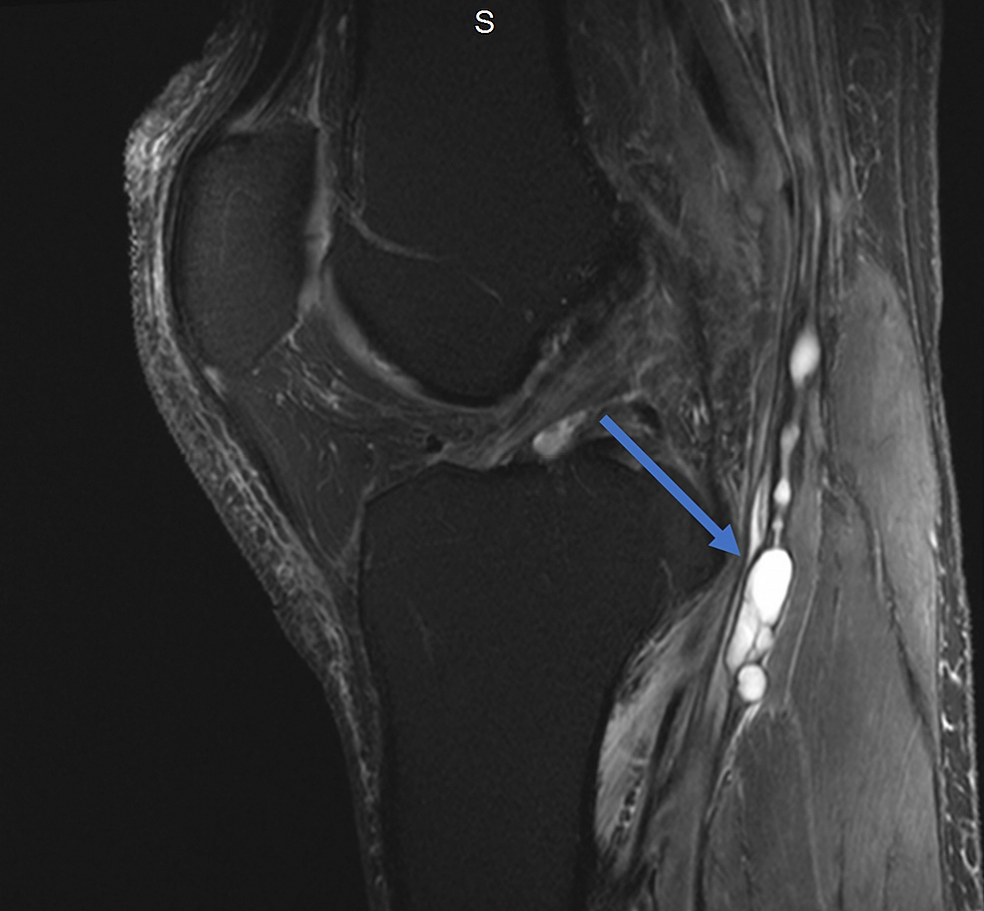
Sagittal T2-weighted TSE fat-saturated image (transverse view) demonstrating signal hyperintensity due to multilobulated tubular cysts within the tibial nerve TSE, turbo spin echo

**Figure 4:**
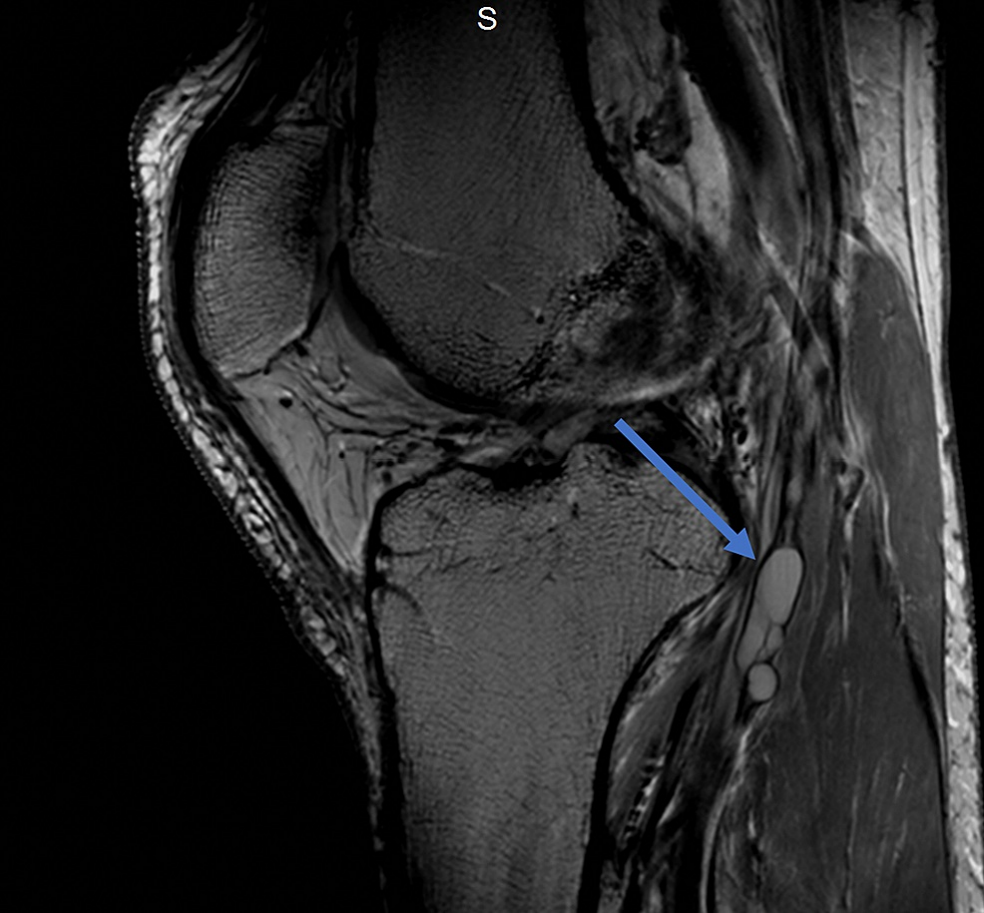
Sagittal PD TSE image demonstrating multilobulated cysts involving the tibial nerve PD, proton density; TSE, turbo spin echo

**Figure 5:**
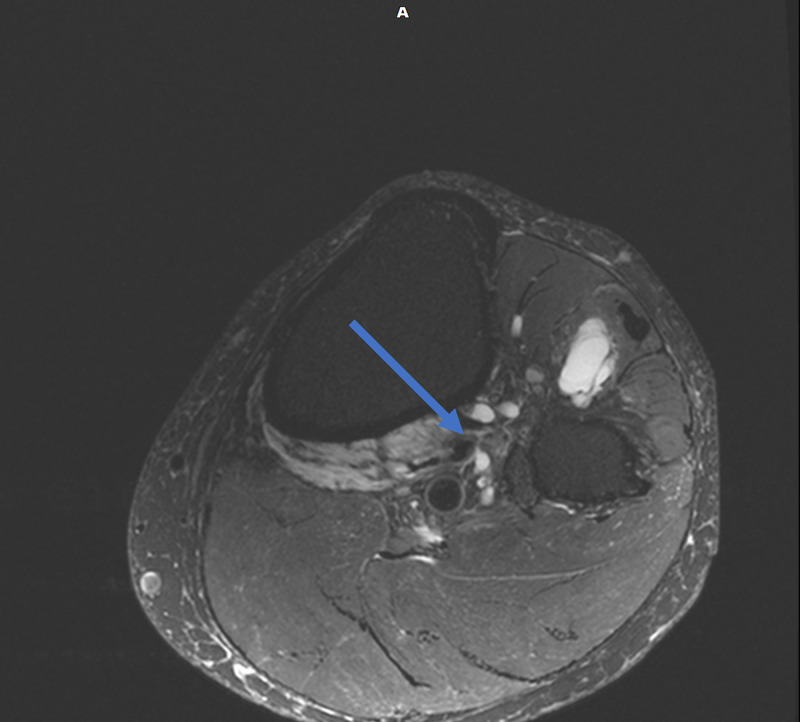
Axial T2-weighted TSE fat-saturated image demonstrating cysts extending to the muscular branch of the popliteus muscle with denervation edema TSE, turbo spin echo

**Figure 6:**
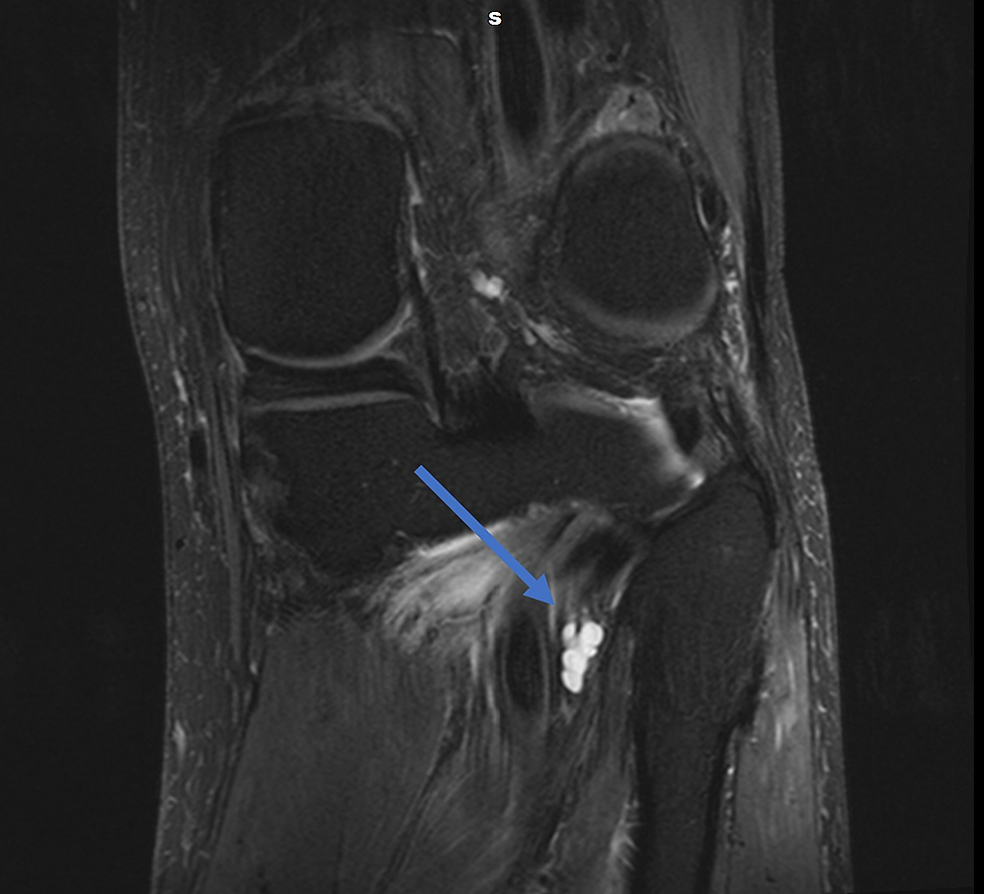
Coronal T2-weighted TSE fat-saturated image demonstrates demonstrating tubular cysts that extend into the popliteus tendon TSE, turbo spin echo

Magnetic resonance imaging (MRI) of the lumbar spine was obtained and showed no evidence of disc herniation, spinal stenosis, or foraminal stenosis. Subsequent MRI of the left knee showed markedly complex cysts at the level of the superior tibiofibular joint. The cysts appeared to connect to the posteromedial superior tibiofibular joint and descend in close proximity to the anterior tibial artery and anterior tibial veins, as appears on the axial and coronal T2-weighted fat-saturated images (Figures 1, 2). Sagittal T2-weighted fat-saturated image demonstrated signal hyperintensity due to multilobulated tubular cysts within the tibial nerve, extending from the posterior tibial epiphysis to the distal femoral epiphysis (Figure 3). Sagittal proton density image demonstrated multilobulated cysts extending within the tibial nerve shealth (Figure 4). Axial T2-weighted fat-saturated image at the level of the posterior medial fibular joint demonstrated intraneural ganglion cysts extending to the muscular branch of the popliteus muscle with denervation edema (Figure 5). Coronal T2-weighted fat-saturated image demonstrated a small amount of tubular cysts that extended into the popliteus tendon at the level of the posterior tibiofibular joint (Figure 6). An extraneural ganglion cyst within the extensor digitorum muscle with thickening of the extensor digitorum tendon was also identified.

Given the findings, the patient was further referred for both orthopedic and neurosurgical consultations. Surgical intervention was discussed, and options included cyst decompression, joint connection-disconnection, and joint resection. The patient opted for indirect decompression through joint resection. He subsequently underwent arthrotomy of the proximal tibiofibular joint with excision of the medial aspect of the proximal fibula including the articular surface of the proximal fibula. The lateral aspect of the articular surface of the tibia was also removed. Resection of the left superior tibiofibular joint was performed with reconstruction of the proximal tibiofibular joint to increase stability. Exploration and mobilization of the left peroneal nerve at the fibular neck region and drainage of anterior compartment cyst were also performed.

On postoperative three-month follow-up, the patient noted improvement of motor strength with minimal footdrop. There was no improvement of sensation on the distal plantar surface of the left foot. Denervation of the posterior tibialis and popliteus muscle was still present. On 12-month follow-up, he was noted to have no additional improvement of motor strength. No improvement of sensation or innervation of the posterior tibialis or popliteus muscles.

## Discussion

Intraneural ganglion cysts are a rare occurrence. They are typically found on the radial, ulnar, median, tibial, and posterior interosseous nerves, most frequently involving the common peroneal nerve [1]. Common clinical presentations include posterior knee and calf pain resulting from tibial neuropathy, with preferential degeneration of the popliteus muscle. Symptoms include pain, paresthesia, and decreased strength that originates in the knee and commonly extends to the plantar surface of the foot. These findings can be mistaken for lumbar neuropathies and compression of the sacral nerve roots [3-8]. Differential diagnosis includes peripheral nerve sheath tumors, periosteal ganglions, Baker’s cysts, extraneural ganglion cysts, and atypical vascular or lymphatic malformations [9-18].

The origin of ganglion cysts is mostly unknown, but many theories exist. A few theories have tried to explain the origin of ganglion cyst citing chronic stress or joint stress causing a rent in the capsular space or mucoid degeneration leading to fluid accumulation [10]. Currently, the most widely accepted and affirmed theory is the unifying theory. The unifying theory hypothesizes that intraneural ganglion cysts originate in nearby joints; the cystic fluid from the synovial joints (through capsular defect) dissect intra-epineuronally from the joint through articular branches perforating the joint capsule along the path of least resistance. In the case of tibial intraneural ganglion cysts, the cysts arise from the posterior aspect of the superior tibiofibular joint unlike anterior cysts, which arise from the peroneal nerve [9,12,14,16].

For the diagnostic process, it has been shown that MRI and ultrasonography are the most useful tools to identify intraneural ganglion cysts. On ultrasonography, an intraneural ganglion cyst will appear as a large, well-circumscribed hypoechogenic lesion. On MRI, it appears as a multilobulated lesion with low signal intensity on T1-weighted images and high signal intensity on T2-weighted images, oriented longitudinally along the course of the affected nerve [7]. MRI findings include the “tail sign”, which is a narrow neck or pedicle connecting the intraneural cyst to the joint with denervation of the popliteus muscle and atrophy. The findings of eccentric displacement of the tibial nerve fascicles by intraneural cysts are referred to as the “popliteus sign” or the “signet ring sign” [6,13,15]. Muscle denervation due to edema can be seen on T2-weighted images as hyperintensity. Muscle atrophy is also characterized by hyperintensity on T1-weighted images [6,17,19]. Articular joint connections are a possible finding but are difficult to detect on MRI or ultrasound.

Treatment of an intraneural ganglion cyst includes surgical excision, decompression, or minimally invasive decompression through percutaneous aspiration of the ganglion under ultrasound guidance [7,8], although these methods have reported recurrences with rates as high as 50-70% [14]. Cases refractory to minimally invasive percutaneous aspiration should follow up with surgical resection. Surgical resection is considered a more definitive treatment but is more challenging and invasive with higher rates of complications such as nerve damage, further muscle atrophy, and infection. Surgery should be performed to resect the entire cyst to prevent recurrence. Articular connections, branches, and synovium should be identified during surgery and eliminated to reduce the possibility of recurrence [3,4,14,17]. In accordance with the articular theory and current practices, Spinner et al. suggest the four-dimensional technique, which includes dissection of the nerve, disarticulation of the tibiofibular joint, decompression of the cyst, and disconnection of the articular branch to treat articular branch connections [18]. Failure to disconnect the articular branch can result in cyst persistence and recurrence, and incomplete resection of the synovium can result in extraneural occurrences [9,20].

## Conclusions

We presented a rare case of intraneural ganglion cyst arising from the tibiofibular joint in a healthy 61-year-old male who was previously in good health and ran frequently. He underwent indirect decompression through joint resection. Unfortunately, he did not have clinical improvement on one-year follow-up. Overall, symptomatic treatment of intraneural ganglion cyst includes decompression, surgical excision, or minimally invasive decompression by percutaneous aspiration of the ganglion under ultrasound guidance.
